# Supplementary material for: Revealing the mechanism of cold metal transfer
Source: Commun Eng. 2025 Mar 5;4:39. doi: 10.1038/s44172-025-00378-6 (PMC11882817; doi:10.1038/s44172-025-00378-6)
Supplement: Supplementary file 2 — Supplementary Information [file 44172_2025_378_MOESM2_ESM.pdf]

Supplementary Information

Revealing the mechanism of cold metal transfer

J. Karimi[1,2], C. Zhao[3]

[1]*Faculty of Production Engineering, University of Bremen, Badgasteiner Str. 1, Bremen 28359, Germany*

[2]*Institute of Materials Engineering, Technische Universität Bergakademie Freiberg, Gustav-Zeuner-Str.5, Freiberg, 09599, Germany*

[3]*School of Materials Science and Engineering, Huazhong University of Science and Technology, Wuhan 430074, China*

.

AA5183 wire and TiC nanoparticles

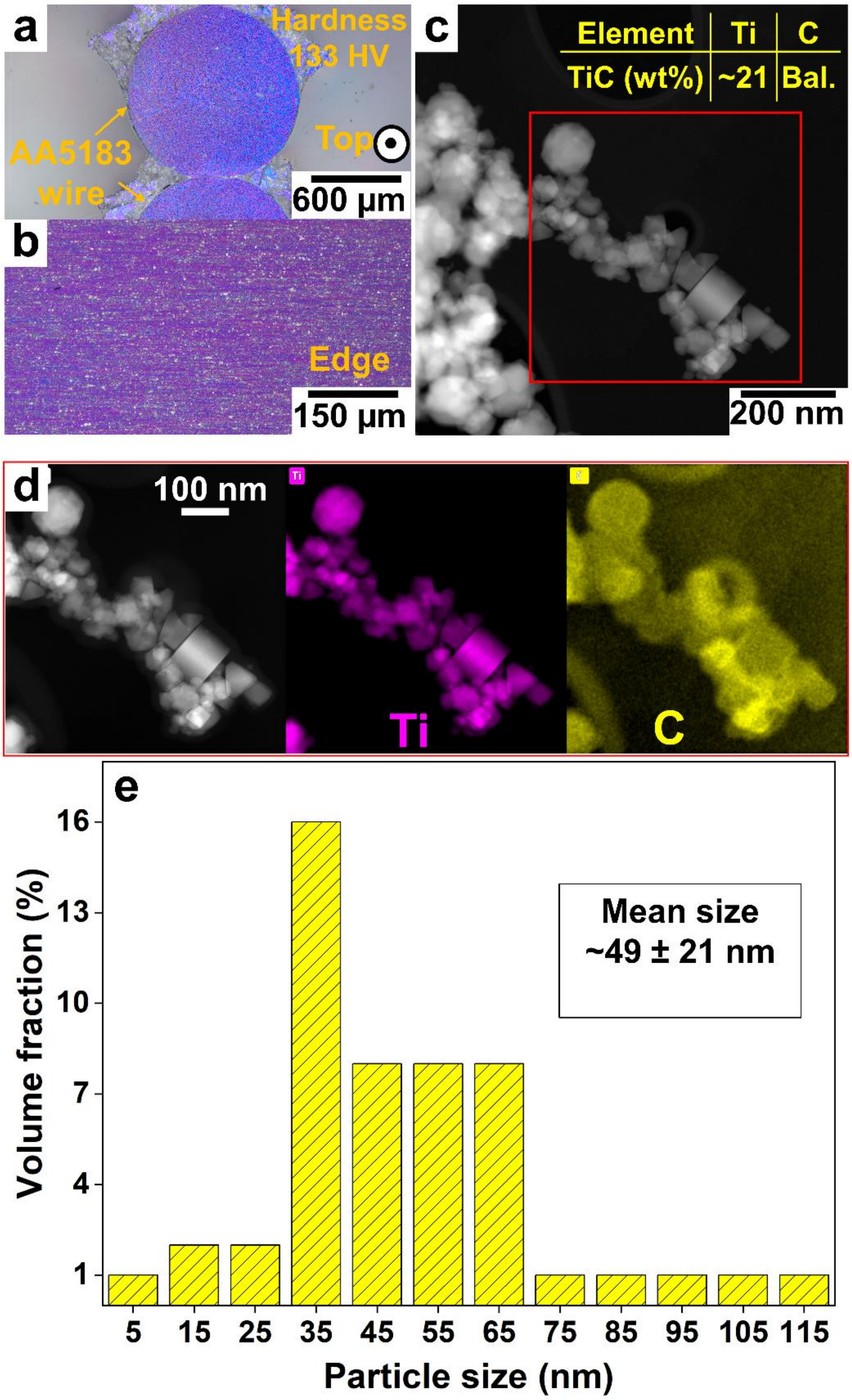

Fig. S1: Microstructure of AA5183 wire in **a** top and **b** edge. **c** TEM of TiC. nanoparticle. The elemental mapping and size distribution of TiC nanoparticles in **d** and **e**, respectively.

Analysis of the microstructure and chemical composition

The chemical composition of wire, and AM parts (pure AA5183 and with the addition of TiC) using EDS in SEM are shown in Table S1. The chemical composition of the AM part with 10 wt% of TiC is selected. All additively manufactured parts showed close values to the nominal compositions. The microstructure of the CMT-WAAM of AA5183 parts was investigated, and showed a fine equiaxed grain morphology. Zhang et al. [1] also investigated the microstructural properties of CMT-WAAM AA5183 and reported fine and equiaxed grain.

Table S1 Chemical composition of the wire, and WAAM-CMT samples with and without TiC nanoparticles

		Element wt%								
		Mg	Fe	Si	Cu	Zn	Ti	Cr	Mn	Al
Wire		4.55±0.26	0.63±0.84	0.1±0.02	0.3±0.36	0.01±0.02	0.05±0.09	0.76 ±1.0	0.64±0.52	Bal.
WAAM-TiC wt%	0	4.6±0.3	0.6±0.8	-	-	0.1±0.1	0.0±0.0	0.8 ±1.0	0.4±0.5	Bal.
	10	5.19±3.05	-	-	-	-	5.86±1.42	-	-	Bal.

Elemental mapping of WAAM part

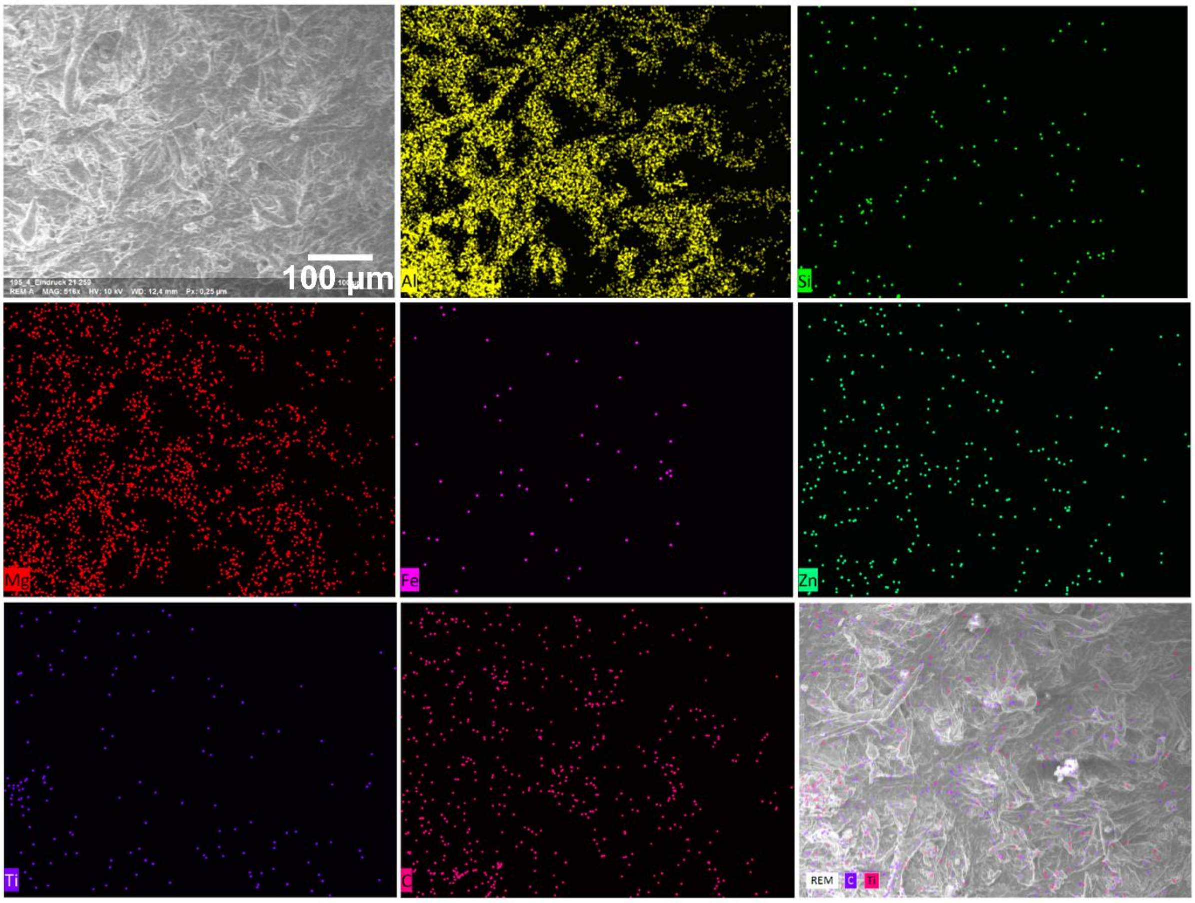

Fig. S2: The elemental mapping of WAAM AA5183 with the addition of 10 wt% TiC.

X-ray diffraction of the WAAM parts

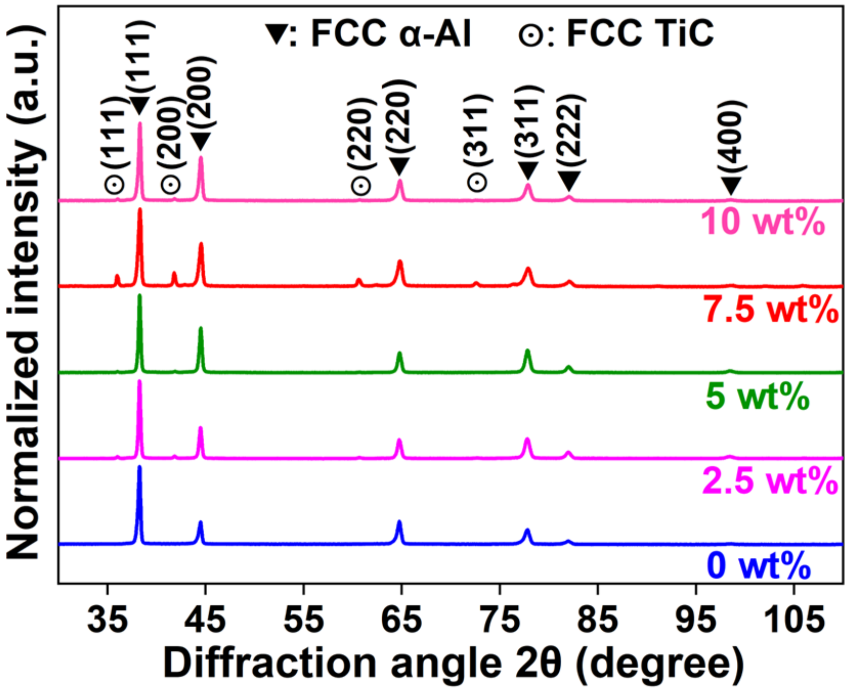

Fig. S3: The X-ray diffraction of the WAA5183 with the different content of TiC nanoparticles.

Density measurement of WAAM parts

The density of the fabricated parts was measured, and the results are shown in Table S2.

Table S2 Measured density of WAAM-CMT samples

	TiC (wt%)				
	0	2.5	5	7.5	10
Density (g/cm^3)	2.63±0.0008	2.45±0.0007	2.38±0.002	2.45±0.017	2.41±0.004
Density (%)	98.69±0.03	90.12±0.02	85.87±0.09	86.61±0.6	83.61±0.15

Reference

[1] Zhang, B., Zhang, L., Wang, C., Wang Z. & Gao, Q. Microstructure and Properties of Al Alloy ER5183 Deposited by Variable Polarity Cold Metal Transfer. *J. Mater. Process. Technol.* **267,** 167–76 (2019).
