# Supplementary material for: Revealing the mechanism of cold metal transfer
Source: Commun Eng. 2025 Mar 5;4:39. doi: 10.1038/s44172-025-00378-6 (PMC11882817; doi:10.1038/s44172-025-00378-6)
Supplement: Supplementary file 3 — Description of Additional Supplementary Files [file 44172_2025_378_MOESM3_ESM.pdf]

Description of Additional Supplementary Files

File name: Supplementary Video 1

Description: The dynamic behaviour of molten materials during wire withdrawal

File name: Supplementary Video 2

Description: The dynamic behaviour of molten materials during wire dipping

File name: Supplementary Video 3

Description: Material ejections from the melt pools for pure AA5183

File name: Supplementary Video 4

Description: Material ejections from the melt pools for AA5183 with TiC nanoparticle additions
